# Identification of gp96 as a Novel Target for Treatment of Autoimmune Disease in Mice

**DOI:** 10.1371/journal.pone.0009792

**Published:** 2010-03-23

**Authors:** Jung Min Han, Nam Hoon Kwon, Jin Young Lee, Seung Jae Jeong, Hee Jung Jung, Hyeong Rae Kim, Zihai Li, Sunghoon Kim

**Affiliations:** 1 Center for Medicinal Protein Network and Systems Biology, College of Pharmacy, Seoul National University, Seoul, Republic of Korea; 2 Department of Molecular Medicine and Biopharmaceutical Sciences, Graduate School of Convergence Science and Technology, Seoul National University, Suwon, Republic of Korea; 3 Research Institute of Pharmaceutical Sciences, College of Pharmacy, Seoul National University, Seoul, Republic of Korea; 4 Cancer & Infectious Disease Research Center, Bio-Organic Science Division, Korea Research Institute of Chemical Technology, Dae Jeon, Republic of Korea; 5 Center for Immunotherapy of Cancer and Infectious Diseases, University of Connecticut School of Medicine, Farmington, Connecticut, United States of America; Centre de Recherche Public de la Santé (CRP-Santé), Luxembourg

## Abstract

Heat shock proteins have been implicated as endogenous activators for dendritic cells (DCs). Chronic expression of heat shock protein gp96 on cell surfaces induces significant DC activations and systemic lupus erythematosus (SLE)-like phenotypes in mice. However, its potential as a therapeutic target against SLE remains to be evaluated. In this work, we conducted chemical approach to determine whether SLE-like phenotypes can be compromised by controlling surface translocation of gp96. From screening of chemical library, we identified a compound that binds and suppresses surface presentation of gp96 by facilitating its oligomerization and retrograde transport to endoplasmic reticulum. *In vivo* administration of this compound reduced maturation of DCs, populations of antigen presenting cells, and activated B and T cells. The chemical treatment also alleviated the SLE-associated symptoms such as glomerulonephritis, proteinuria, and accumulation of anti-nuclear and –DNA antibodies in the SLE model mice resulting from chronic surface exposure of gp96. These results suggest that surface translocation of gp96 can be chemically controlled and gp96 as a potential therapeutic target to treat autoimmune disease like SLE.

## Introduction

SLE is a systemic autoimmune disease characterized by abnormalities in dendritic cell (DCs), autoreactive T cells and B cells [1],[2]. DCs are important in regulating both immunity and tolerance and have been implicated in the pathogenesis of SLE [1]. DCs induce activation of naïve T cells and stimulate B cell growth and differentiation. Therefore, lupus-associated DCs producing altered signals and amplifying autoreactive specificities in T cells, which, in turn, provide help to autoreactive B cells, inducing an increase in autoantibody production. Glomerulonephritis is induced when DNA specific autoantibodies form complexes in kidney glomerulus [3],[4]. As disease progresses, mesangial proliferation, endocapilliary proliferation, vascular collapse and immune complex accumulation in kidney result in glomerulonephritis and eventual renal failure [3],[4]. SLE is treated by immunosuppresants and cytostatic agents, with extensive use of corticoids when disease is stabilized, but these treatments have numerous side effects [5].

Gp96 is the endoplasmic reticulum (ER)-resident chaperone protein belonging to the HSP90 family [6]. The continuous recycling of escaped ER resident proteins such as gp96, GRP78/Bip, protein disulfide isomerase (PDI), and calreticulin is mediated by retrograde transport form Golgi to ER through COPI-coated vesicles [7] [Bibr pone.0009792-Letourneur1] [–] [10]. ER localization of these proteins is regulated through their C-terminal KDEL sequence. KDEL sequence is recognized by the KDEL receptor ERD2 [11], which is mainly localized to the cis-Golgi [7],[12]. Binding of KDEL proteins to ERD2 leads to its oligomerization [13] and stimulates its rapid transport out of cis-Golgi [14],[15]. Oligomerization seems to be a hallmark of constitutively cycling proteins of the early secretory pathway, as ERGIC-53 is a stable hexamer [16] and the KDEL receptor oligomerizes upon binding to its ligands [13]. gp96 also exists as a homodimer or higher-order oligomer [17]–[19], although it is not known whether dimer or higher-order oligomer is responsible for ERD2 binding. The ERD2-gp96 complex returns to the ER where it dissociates, thus freeing ERD2 for further cycling of transport.

In addition to the intracellular chaperone function, gp96 has been implicated in innate and adaptive immunity [20],[21] and its cell surface exposure is associated with its immunological activities such as the activation or maturation of dendritic cells (DCs) [22]–[24]. Direct interaction between gp96 and DCs via CD91 and TLR2/4 [25]–[27] induces DC maturation, resulting in proinflammatory cytokine secretion and MHC class I and II upregulation [27],[28]. Transgenic mice chronically expressing gp96 on cell surfaces showed significant DC activation and spontaneous systemic lupus erythematosus (SLE)-like autoimmune phenotypes [29]. Gp96 is increased in synovial fluid from the joints of human rheumatoid arthritis patients and the expression of gp96 shows a correlation with inflammation and synovial lining thickness, further supporting the pathological association of gp96 with autoimmune diseases [30].

AIMP1/p43 (ARS-interacting multi-functional protein 1, also known as p43) is a protein involved in diverse physiological processes [31]. Recently, we found that AIMP1 holds gp96 in ER, preventing its extracellular translocation [32]. For this reason, AIMP1-deficient mice contain cells with increased surface levels of gp96, thereby displaying the phenotypes similar to those of gp96tm transgenic mice [32]. Although all of these previous studies demonstrated the importance of the ER retention of gp96 to prevent aberrant autoimmune responses, it is not yet determined whether SLE-like phenotypes resulting from chronic exposure of gp96 can be compromised by suppressing its surface translocation. To address this question, we first screened chemical library to identify a compound that can bind and blocks surface localization of gp96. After determining the mode of action of the selected compound upon binding to gp96, we evaluated its *in vivo* effect on various phenotypes that are associated with SLE symptoms using the transgenic mice displaying SLE-like phenotypes resulting from the chronic surface presentation of gp96.

## Results

### Screening and Identification of GPM1 as a gp96-Binding Chemical

Since AIMP1 interacts with gp96 and regulates its ER localization, preventing cell surface localization [32], we hypothesize that small molecule, having similar activity like AIMP1, can suppress cell surface localization of gp96 and aberrant autoimmune responses although small molecule may compete with AIMP1 for gp96 binding. For this, we set up modified ELISA assay method using recombinant AIMP1 and gp96 proteins. The screening assay was designed to select the compounds that block the interaction of gp96 with AIMP1 as described in Methods. In the chemical screening, we chose 6,482 representative chemicals with different pharmacophore representing 150,000 chemicals deposited in Korea Chemical Bank (http://www.chembank.org/).

**Figure 1:**
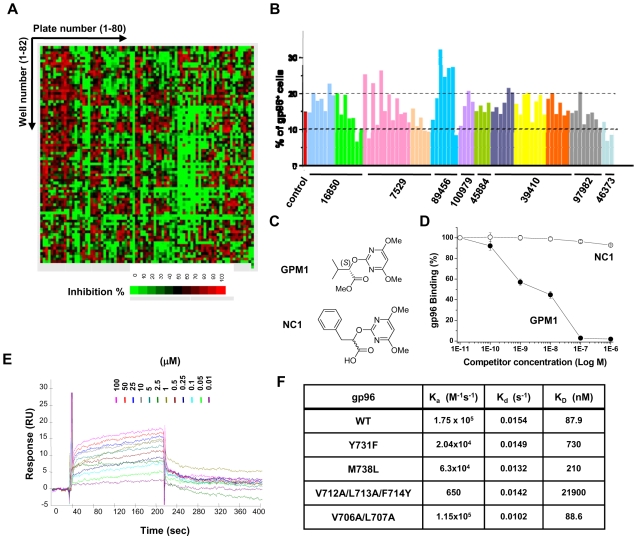
Identification of gp96-binding compound that suppresses plasma membrane levels of gp96. (A) Heat map of primary screening data. Inhibitory effects of 6,482 chemicals on the interaction of gp96 and AIMP1 were monitored by modified ELISA method as described. Taking the value of DMSO as 0%, the inhibition of each chemical was indicated as relative percentage and the degree of the inhibition was represented by a heat map in a 10% scale from 0 (green) to 100% (red). (B) Effect of the derivatives of the primary hits on surface expression of gp96. RAW264.7 cells were treated with 77 compounds (10µM, 24h) and the effects on surface expression of gp96 were analyzed by flow cytometry. We took 5% changes compared to the control group (15% of the gp96^+^ cells) as cut-off values. Out of 77 compounds, 8 compounds showed suppressive effect on surface expression of gp96 below 10% whereas 12 compounds showed increased gp96^+^ cells more than 20%. The numbers indicate the primary hits (see table S1) and the effects of their derivatives are shown as bar graphs. The compounds in the same plate are displayed as one color. (C) Chemical structure of GPM1 and negative control (NC1) used in this study. (D) The dose-dependent effect of GPM1 or NC1 on the AIMP1-gp96 interaction using ELISA method as described. The indicated concentrations of the two compounds and biotin-conjugated murine gp96 were added to AIMP1 coated on the surface of microtiter wells, and gp96 bound to AIMP1 was detected with strepavidin-conjugated peroxidase. (E) The binding of GPM1 to gp96 was examined by surface plasmon resonance (SPR). GPM1 at the indicated concentrations was injected to immobilized gp96 and the binding was measured using Biacore 3000. The response data were processed using data from a reference surface and buffer injections. Each concentration was tested in triplicate. (F) Association rate constant (K_a_), dissociation rate constant (K_d_), and equilibrium dissociation constant (K_D_) were determined for the interactions of GPM1 with various mutants of gp96 by the SPR experiments as described in Methods.

Each of the tested compounds gave different inhibitory effect on the interaction of the two proteins (Fig. 1A), and we have selected 12 compounds that inhibited the interaction more than 95% of the control at 0.1µM (Table S1). These 12 compounds had no effect on the interaction between lysyl-tRNA synthetase (KRS) and superoxide dismutase 1 (SOD1) [33] indicating the specificity of compounds. We obtained 1251 additional derivative compounds from the 12 initial hits and tested them again for the activities against the gp96-AIMP1 interaction. Among them, 77 compounds showed negative effect while 1174 compounds gave no effect (Table S1). The chemicals that would block the interaction of gp96 and AIMP1 are expected to either enhance or suppress the surface localization of gp96 depending on their binding sites. If chemicals bind to AIMP1 side, they are expected to enhance the surface localization of gp96 since it would release gp96 from its intracellular anchor, AIMP1. But if chemicals bind to gp96 side, they may suppress the surface translocation of gp96 by interfering with the subsequent processes for the molecular trafficking of gp96 to the plasma membrane. We thus examined the effects of the 77 compounds on the surface expression of gp96 by flow cytometry as described. Out of 77 compounds, 8 reduced gp96 positive cells to below 10% whereas 12 compounds increased gp96 positive cells to more than 20% and the rest 57 compounds gave the effects within the range between them (Fig. 1B and Table S1). Among the 8 selected compounds, four were discarded for the following experiments due to their cellular toxicity (data not shown). In the next step, we subjected the remaining 4 compounds to the single dose toxicity test (single i.p. injection, 500mg/kg, 1 week) using mice. Among them, [(S)-methyl 2-(4,6-dimethoxypyrimidine-2-yloxy)-3-methylbutanoate] (designated as GPM1, Fig. 1C upper), showed no toxicity (data not shown) and gave high score in leadlikeness rule [34]. Among other 3 compounds, one gave severe toxicity to mice and the other two showed low lead-likeness score (data not shown). Based on these results, we finally chose GPM1 for further experiments. Among the total screened compounds, 2-(4,6-dimethoxypyrimidin-2-yloxy)-3-phenylpropanoic acid showed only 4% inhibition on the interaction of gp96 and AIMP1 despite it shares dimethoxypyrimidine ring with GPM1. We thus selected it as a control (NC1) (Fig. 1C lower).

We compared the dose-dependent effect of GPM1 and NC1 on the interaction of gp96 and AIMP1. GPM1 showed 50% inhibition at around 30nM whereas NC1 gave no effect on the binding of the two proteins (Fig. 1D). We monitored the binding kinetics of GPM1 to gp96 or AIMP1 by surface plasmon resonance (SPR) using BIAcore 3000. GPM1 showed the binding to gp96 with *K*_D_ of 87.9nM (Fig. 1E and F), but no apparent binding to AIMP1 (data not shown). We also determined whether GPM1 can bind to protein disulfide isomerase (PDI) and GRP78/Bip that are also an ER-resident KDEL protein like gp96. GPM1 showed extremely weak binding to PDI and GRP78/Bip compared to gp96 (*K*_D_ = 249 µM and 11.9 mM, respectively) (Fig. S1A and S1B). To further define the GPM1-binding region of gp96, we introduced point mutations at L707, V706, V712, L713, F714, Y731 and M738 that are located within AIMP1-binding region of gp96 [32] and monitored how the mutations at these sites would affect the binding of GPM1 to gp96 using SPR method. All the mutants retained the normal ATPase activity of gp96 (Fig. S2), suggesting that the mutations did not affect their native conformation. However, GPM1 showed significantly decreased affinity to the mutants except for V706A/L707A mutant, suggesting the importance of the C-terminal dimerization domain of gp96 for the binding to GPM1.

### Suppression of Cell Surface gp96 by GPM1 through Increased ER Retention

**Figure 2:**
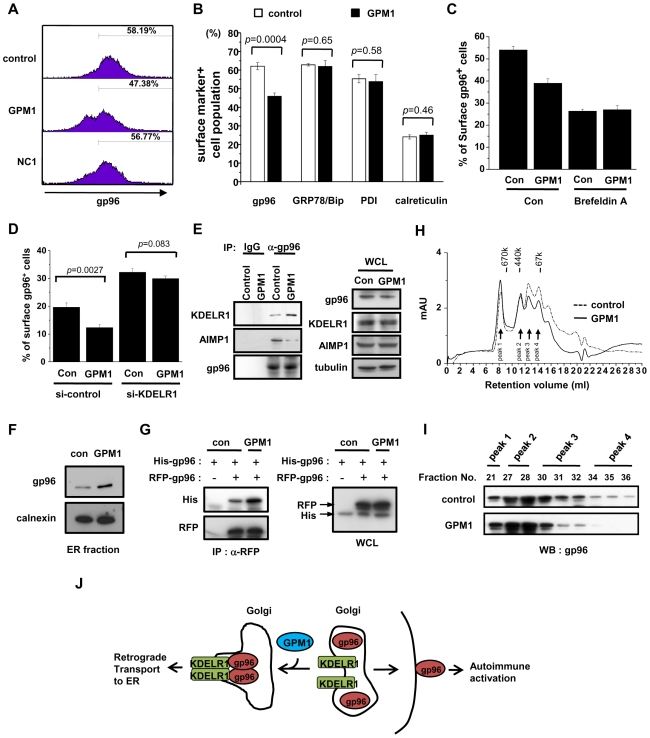
The effect of the selected chemical on gp96 oligomerization and surface levels of gp96. (A) Splenocytes isolated from C57BL/6 mice were treated with vehicle (5% DMSO in PBS), GPM1, or NC1 (10µM, 24h), stained for cell surface gp96, and followed by flow cytometry. The numbers indicate the percentage of gp96^+^ splenocytes. (B) The effect of GPM1 on cell surface gp96, GRP78/Bip, PDI, and calreticulin. RAW264.7 cells were treated with DMSO or GPM1 (10µM) for 24h. Cells were stained for cell surface gp96, GRP78/Bip, PDI, and calreticulin, followed by flow cytometry. (C) RAW264.7 cells were treated with DMSO, GPM1 (10µM), or brefeldin A (1µg/ml) for 4h. Cells were stained for cell surface gp96, followed by flow cytometry. (D) RAW264.7 cells were transfected with si-control or si-KDELR1 for 48h and then further treated with DMSO or GPM1 (10µM) for 24h. Cells were stained for cell surface gp96, and followed by flow cytometry. (E) RAW24.7 cells were treated with 10µM GPM1 for 24h. Cell lysates were immunoprecipitated with control IgG or anti-gp96 antibody and the immunoprecipitated proteins were immunoblotted with anti-KDELR1, anti-AIMP1 and gp96 antibodies. WCL means whole cell lysate. (F) RAW264.7 cells were treated with 10µM GPM1 for 24h. Cell lysates were fractionated using sucrose density gradient. Proteins from ER fraction were immunoblotted with anti-gp96 antibody. Calnexin was used as an ER marker. (G) The effect of GPM1 on gp96 oligomerization. 293 cells were transfected with RFP-tagged gp96 and His-tagged gp96. Cells were then treated with DMSO or GPM1 (10µM) for 4h. Cell lysates were immunoprecipitated with anti-RFP antibody, and the precipitated proteins were immunoblotted using anti-His antibody. WCL, whole cell lysates. (H) FPLC gel filtration chromatography of gp96 protein. The purified gp96 (5µM) was incubated with DMSO or GPM1 (50µM) for 1h on ice and then injected into a Superdex 200 10/300 GL size exclusion column. The positions of mass standards used for calibration are indicated at the top of the chromatogram. Molecular weight: thyroglobin (670 kDa), ferritin (440kDa) and BAS (67kDa). Peak 1, 2, 3, and 4 indicate the position of oligomer, tetramer, dimer, and monomer of gp96 protein, respectively. (I) Peak fractions from gel filtration of DMSO or GPM1-treated gp96 proteins were immunoblotted with anti-gp96 antibody. (J) Model for GPM1 action. In the absence of KDEL proteins such as gp96, KDEL receptor exists mainly in monomeric form. GPM1 treatment leads to gp96 oligomerization and binding to KDEL receptor, which induces retrograde transport of gp96 to ER. The data represent the means ± S.D. Student t-test *P* values are shown.

First, we tested the effect of GPM1 on the surface localization of gp96. We isolated splenocytes from C57BL/6 mice, treated with vehicle, GPM1, or NC1 (10µM, 24h), and monitored the surface levels of gp96 by flow cytometry. The surface gp96 levels in GPM1-treated splenocytes were lower compared to those of the vehicle- or NC1-treated cells (Fig. 2A). We also determined whether GPM1 can affect the cell surface level of GRP78/Bip, PDI, and calreticulin that are also an ER-resident KDEL protein like gp96. GPM1 had no effect on the populations of the surface GRP78/Bip, PDI, and calreticulin positive cells while it reduced gp96-positive cell population, suggesting the specificity of GPM1 for gp96 (Fig. 2B). Next, we examined the action mechanism of GPM1 for suppression of cell surface gp96. Since GPM1 has no effect on the cellular protein level of gp96, GPM1 is expected to have two action points. The first possibility is that GPM1 directly enhances the endocytosis of cell surface gp96 and the second possibility is that GPM1 enhances the retrograde transport from Golgi to ER, resulting in the decreased movement of gp96 to the cell surface. Since surface expression of gp96 was abrogated in the presence of brefeldin A (BFA), which leads to Golgi disturbance and protein accumulation inside the ER [35], we monitored the effect of GPM1 on the cell surface gp96 in the absence of the translocation of gp96 from the Golgi to the cell surface by BFA treatment. If GPM1 has an effect on endocytosis of cell surface gp96, it should additionally suppress cell surface gp96 in the presence of BFA. But, GPM1-induced suppression of surface gp96 was abrogated in the presence of BFA (Fig. 2C), suggesting that the target point of GPM1 is the translocation of gp96 from the ER to the cell surface. To determine the functional importance of KDELR1 for the suppressive effect of GPM1 on the surface localization of gp96, we introduced siRNA to knockdown KDELR1 transcript and examined how GPM1 would affect the surface levels of gp96. In the control cells, GPM1 reduced gp96 positive cell population (Fig. 2D). When the cells were transfected with KDELR1 siRNA, GPM1 effect was ablated while gp96 positive cells were increased due to the depletion of KDELR1 (Fig. 2D). These results demonstrate that the suppressive effect of GPM1 on the surface translocation of gp96 requires KDEL receptor. We tested whether GPM1 would affect the binding of gp96 to KDEL receptor 1 (KDELR1) by co-immunoprecipitation between the two proteins. GPM1 increased the amount of gp96 bound to KDELR1 while blocking the binding of gp96 to AIMP1 (Fig. 2E left). However, GPM1 did not decrease the cellular levels of three proteins (Fig. 2E right) and the ATPase activity of gp96 (Fig. S3A). The ER retention of gp96 is enhanced via its interaction with AIMP1 that facilitates the oligomerization of gp96 [32]. We thus examined whether GPM1 would functionally mimic AIMP1 for the ER residency of gp96. When we isolated ER fraction from control-treated or GPM1-treated cells, the ER residency of gp96 was increased by GPM1 treatment (Fig. 2F). The retrograde transport of gp96 from Golgi to ER is mediated through the recognition of the C-terminal KDEL motif of gp96 by the KDEL receptor [11]. Binding of gp96 to KDEL receptor leads to its oligomerization [13] and retrieval to ER [14]. Thus, oligomerization status of gp96 can be a signature for its ER residency. Indeed, GPM1 treatment increased the amount of His-tagged gp96 coimmunoprecipitated with RFP-tagged gp96 (Fig. 2G), suggesting that GPM1 can regulate gp96 oligomer formation. When we monitored the conformational change of gp96 induced by GPM1, the α-helicity of gp96 was slightly increased as shown by circular dichroism spectroscopy (Fig. S3B). Here we examined whether GPM1 would directly affect oligomerization of gp96 using size exclusion chromatography. When the purified gp96 was mixed with GPM1, the monomer fraction of gp96 was shifted to the oligomers (Fig. 2H and I), suggesting that the oligomerization of gp96 can be facilitated by the binding to GPM1.

Based on these results, we propose the working mechanism of GPM1 as schematically shown in Fig. 2J. Namely, gp96 can be exposed to extracellular space upon various cellular stresses to trigger autoimmune responses. GPM1 binds to the C-terminal dimerization domain of gp96 and enhances oligomerization, leading to the KDEL receptor-mediated retrograde transport to ER.

### Suppression of Cell Surface gp96 by GPM1 Reduces DC Maturation

**Figure 3:**
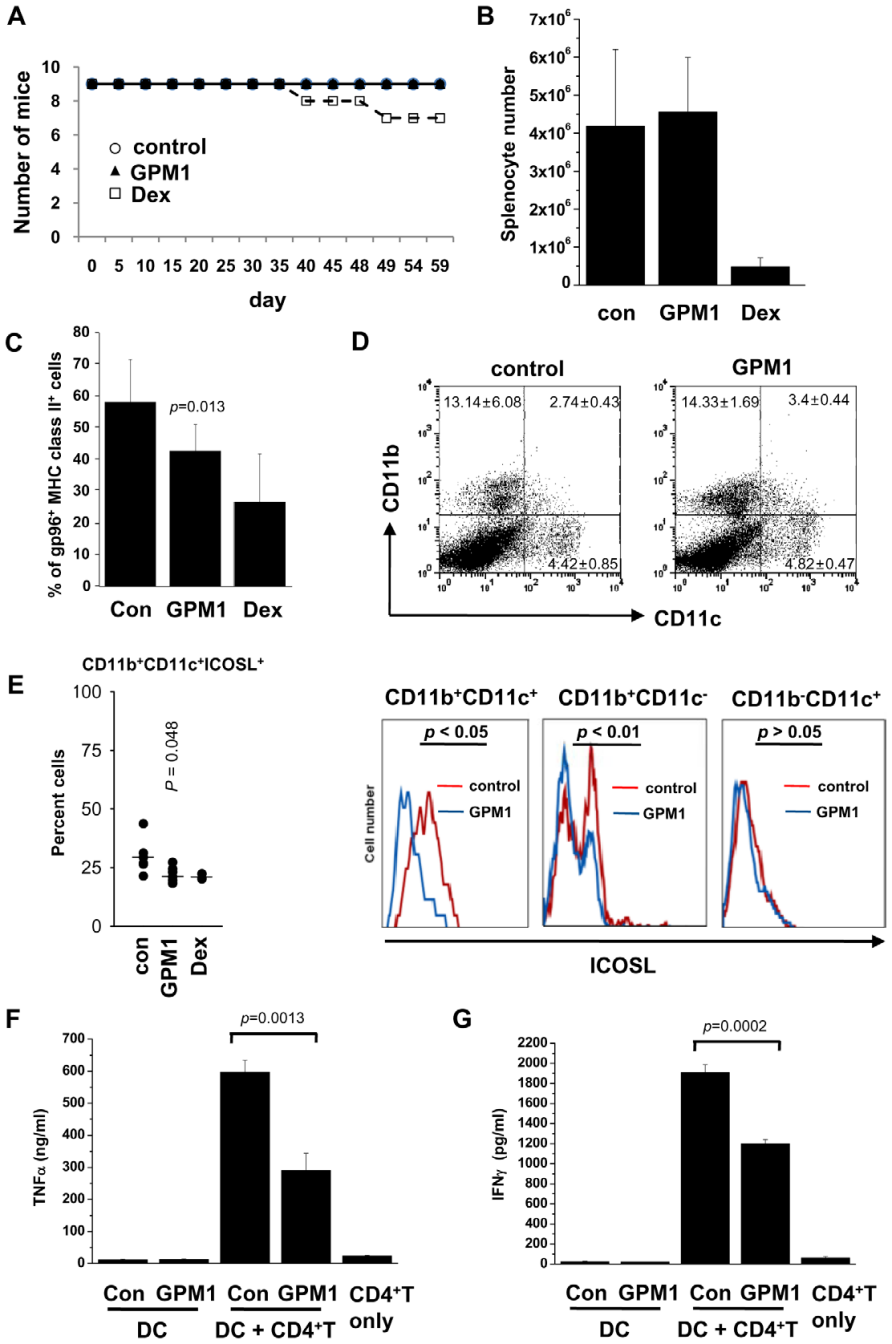
*In vivo* effect of the selected compound on DC maturation. (A) Survival rate of gp96tm transgenic female mice treated with vehicle (n = 9), GPM1 (n = 9), and dexamethasone (n = 9) at 30mg/kg/day. (B) The numbers of total splenocytes isolated from vehicle- (n = 9), GPM1- (n = 9), and Dex (dexamethasone)- (n = 7) treated mice. (C) Percentage of gp96^+^ MHC class II^+^ cell population in splenocytes from gp96tm transgenic female mice treated with vehicle (control) (n = 9), GPM1 (n = 9), and dexamethasone (n = 7) at 30mg/kg. (D) Cell surface ICOSL of CD11b^+^CD11c^+^ (myeloid DCs), CD11b^−^CD11c^+^ (lymphoid DCs), and CD11b^+^CD11c^−^ (macrophages) cell populations in splenocytes from gp96tm transgenic female mice treated with vehicle (n = 9) and GPM1 (n = 9) at 30mg/kg. (E) Cell surface ICOSL^+^CD11b^+^CD11c^−^ cell populations in lymph nodes from gp96tm transgenic female mice treated with vehicle (n = 9) and GPM1 (n = 9) at 30mg/kg. Freshly isolated CD11c^+^ splenic DCs from vehicle- or GPM1-treated mice were tested for their abilities to stimulate allogeneic naïve WT CD4^+^ T cells to produce TNFα (F) and IFNγ (G). Student t-test *P* values are indicated.

*In vivo* effects of GPM1 were determined on cell surface presentation of gp96 and DC maturation associated with SLE. GPM1 was systemically delivered to gp96tm transgenic mice in which the surface levels of gp96 are chronically enhanced [29]. We used phosphate-buffered saline containing 5% DMSO and dexamethasone (Dex), an immunosuppressant of glucocorticoid family as negative and positive control, respectively. Each group consisted of age-matched nine female mice. During intraperitoneal administration for 2 months at the dose of 30mg/kg, GPM1-treated mice did not give apparent adverse effects whereas two of the nine Dex-treated mice died during the treatment (Fig. 3A). After 2 month, we isolated splenocytes from vehicle-, Dex-, and GPM1-treated mice and determined the viability of splenocytes. GPM1 did not affect the viability of splenocytes whereas Dex significantly damaged cell viability (Fig. 3B). We then confirmed the effect of GPM1 on the surface localization of gp96 of splenocytes from vehicle-, dexamethasone-, or GPM1-treated mice. We isolated MHC class II^+^ splenocytes and lymph nodes from vehicle-, Dex-, and GPM1-treated mice and determined the portion of gp96^+^ cells by flow cytometry. The percentages of gp96^+^ MHC class II^+^ cells in the control, Dex- and GPM1-treated groups were about 57, 25 and 41%, respectively, suggesting the suppressive effect of GPM1 on the surface translocation of gp96 (Fig. 3C).

Gp96 has been implicated in the activation and maturation of DCs via CD91 and Toll-like receptor (TLR) 2/4 [25] [–] [27]. Also, DCs from gp96tm transgenic mice and AIMP1-deficient mice were shown to be hyperfunctional [29],[32]. We tested whether suppression of cell surface gp96 through the increase of retrograde transport to ER affected DC maturation in GPM1-treated mice. GPM1-treated mice showed little difference in CD11b^+^CD11c^+^ myeloid DC and CD11b^−^CD11c^+^ lymphoid DC populations (Fig. 3D upper). We monitored the maturity of DCs by measuring the surface levels of inducible costimulator ligand (ICOSL), one of the known DC maturation markers using flow cytometry and found that GPM1 significantly reduced ICOSL levels in splenic CD11b^+^CD11c^+^ myeloid DC and splenic CD11b^+^CD11c^−^ macrophages (Fig. 3D lower), and also reduced lymph node ICOSL^+^CD11b^+^CD11c^+^ myeloid DCs (Fig. 3E). However, GMP1 gave little effect on the ICOSL levels in CD11b^−^CD11c^+^ lymphoid DCs (Fig. 3E right graph). The decreased expression of cell surface ICOSL correlates with the decreased ability of GPM1-treated splenic DCs to activate allogeneic naïve CD4^+^ T cells, as measured by TNFa secretion (Fig. 3F) and IFNγ secretion (Fig. 3G). Based on these results, we conclude that suppression of cell surface gp96 affected DC maturation in GPM1-treated mice.

### Suppression of Cell Surface gp96 by GPM1 Reduces B Cells, Memory T cells in gp96tm Transgenic Mice

**Figure 4:**
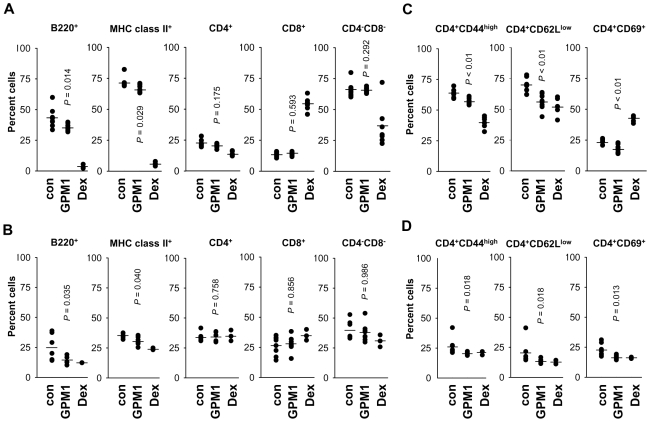
*In vivo* effect of the selected compound on various immune cells. (A) Percentage of B220^+^, MHC class II^+^, CD4^+^, CD8^+^, CD4 and CD8 double negative cell populations in splenocytes from gp96tm transgenic female mice treated with vehicle (n = 9), GPM1 (n = 9), and dexamethasone (n = 7) at 30mg/kg. (B) Percentages of B220^+^, MHC class II^+^, CD4^+^, CD8^+^, CD4 and CD8 double negative cell populations in lymph nodes from gp96tm transgenic female mice treated with vehicle (n = 9), GPM1 (n = 9), and dexamethasone (n = 7) at 30mg/kg. (C) Percentage of CD4^+^CD44^high^ (memory T cells), CD4^+^CD62L^low^ (effector T cells), and CD4^+^CD69^+^ (activated T cells) in splenocytes from gp96tm transgenic female mice treated with vehicle (n = 9), GPM1 (n = 9), and dexamethasone (n = 7) at 30mg/kg. (D) Percentages of CD4^+^CD44^high^, CD4^+^CD62L^low^, and CD4^+^CD69^+^ lymph node cells in vehicle- (n = 9), GPM1- (n = 9), and dexamethasone- (n = 7) treated mice. Student t-test *P* values comparing GPM1-treated mice with control are indicated.

We then examined whether GPM1 could influence the cellular phenotypes that can influence SLE-like phenotypes. The GPM1-treated mice showed reduced populations of B220^+^ and MHC class II^+^ cells in the spleen and in the lymph nodes (Fig. 4A and B left two graphs, respectively). GPM1 gave little effect on the T cell populations in both organs (Fig. 4A and B right three graphs). SLE involves abnormal activation of CD4^+^ T cells that accumulate as activated memory cells and contributes to B cell activation and expansion, and hypergammaglobulinemia [3],[4]. We found a reduction in the population of CD4^+^ memory cells and activated CD4^+^ T cells in the spleen and lymph nodes of GPM1-treated mice (CD4^+^CD44^high^, CD4^+^CD62L^low^, and CD4^+^CD69^+^ cells; Fig. 4C and D). GPM1 treatment also decreased mature B cell population (B220^+^IgM^+^IgD^+^; data not shown).

### Suppression of Cell Surface gp96 by GPM1 Reduces Renal Disease in gp96tm Transgenic Mice

**Figure 5:**
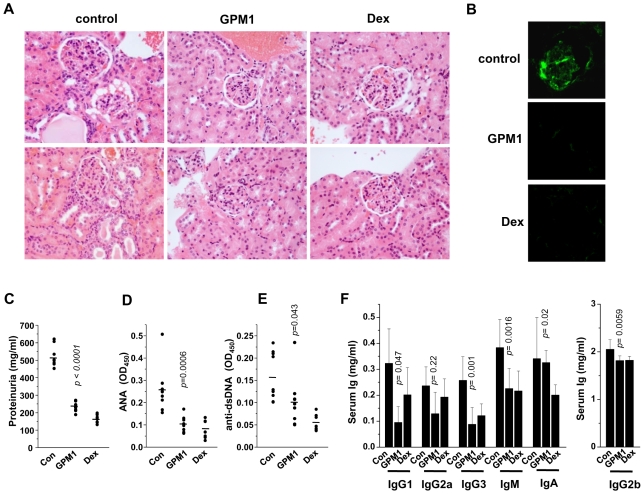
*In vivo* effect of the selected compound on SLE-associated phenotypes in gp96tm transgenic mice. (A) Representative kidney sections stained with hematoxylin and eosin from control (n = 9), GPM1- (n = 9) and dexamethasone (Dex)-treated (n = 7) groups. (B) Glomerular immunoglobulin deposition is shown by green fluorescence with FITC-conjugated goat anti-mouse Ig antibody in vehicle and GPM1-treated kidney. (C) Protein concentrations (mg/ml) in the urines, serum levels of nuclear antigen-specific (D) and double strand DNA-specific antibody (E) were compared between control (n = 9), GPM1- (n = 9) and dexamethasone (Dex)-treated (n = 7) groups. Arbitrary units represent autoantibody absorbance at OD_450nm_ at 1: 101 serum dilution. (F) Serum Ig levels from control (n = 9), GPM1- (n = 9), and Dex-treated (n = 7) groups by ELISA. Total IgG1, IgG2a, IgG2b, IgG3, IgM, and IgA levels were determined using sandwich ELISA kits from Southern Biotechnology Associates (Birmingham, AL). Data represent the mean±S.D. Student t-test *P* values comparing GPM1-treated mice with control are shown.

We checked whether the typical SLE-like phenotypes are also compromised by systemic administration of GPM1. Although vehicle-treated mice displayed severe glomerulonephritis phenotypes such as thickening of basement membrane and capillary walls, vascular obliteration, increased mesangial cells, and renal tubules packed with pink proteinaceous materials (Fig. 5A left column and Table S2) and glomerular immunoglobulin deposition (Fig. 5B top), GPM1 as well as Dex treatment alleviated the symptoms (Fig. 5A and 5B, middle columns). Proteinuria, the sign of kidney dysfunction, the serum levels of nuclear antigen-specific and double stranded DNA-specific autoantibodies were also reduced in GPM1-treated mice (Fig. 5C, D and E, respectively). GPM1 treatment improved hypergammaglobulinemia symptom shown in these mice [29] as determined by total serum levels of IgG1, IgG2b, IgG3, IgM, and IgA (Fig. 5F).

These results show that the selective suppression of cell surface gp96 in gp96tm transgenic mice reduced SLE disease severity. We document possible one mechanism for this recovery, the reduction of DC maturation. These results suggest that the surface gp96 is critical cause of SLE-like disease and may be a useful target in the treatment of SLE.

## Discussion

SLE is a clinically heterogeneous autoimmune disorder with diverse genetic causes [36], and the genes encoding HLA class II, mannose binding protein (MBP), TNFα, the T cell receptor, interleukin 6 (IL-6), CR1, immunoglobulin Gm and Km allotypes, FcgRIIA and FcgRIIIA (both IgG Fc receptors), and heat shock protein 70 are thought to be associated with SLE [37]. Despite the complex molecular etiology of SLE, corticosteroids are commonly used to treat most of the SLE patients but long term usage of these drugs may induce numerous side effects, urging a need to develop more target- or mechanism-based therapy with less adverse effect. In this study, Dex showed several side effects on mouse and splenocyte survival (Fig. 3A and B), and abdominal obesity (data not shown) although Dex reduced phenotypes associated with SLE. However, GPM1 did not show any effect on survival or organ problems (data not shown) according to the long-term treatment. At this moment, we do not yet know how much portion of human SLE results from aberrant localization of gp96, and how the effect of GPM1 is in other spontaneous lupus animal models. These issues are currently under investigation.

The clinical need for novel targets and therapies for human autoimmune disease is high. Previous reports have demonstrated that several novel targets and drug-like compounds for autoimmune diseases are identified. Björk *et al.* identified human S100A9 as a novel target for autoimmune disease via binding to Quinoline-3-carboxamides [38]. Neubert *et al.* reported that proteosome inhibitor bortezomib successfully depletes plasma cells and protects mice from lupus [39]. Barber *et al.* reported that PI3Kγ inhibitor blocks SLE symptoms [40]. Johnson *et al.* reported that mitochondrial F_1_F_0_-ATPase, which is a target of benzodiazepine Bz-423, is a useful target for lupus [41]. In this study, we are suggesting that cell surface gp96 is also candidate target for lupus.

GPM1 directly interacts with gp96 and hydrophobic residues of gp96 within dimerization domain are important to GPM1 binding (Fig. 1E and F). Crystal structure of mammalian gp96 shows that along with the dimerization interface, helix C4 and helix C5 of the C domain of gp96 contains GPM1 binding motifs [18]. In the vicinity of helix C4 and C5, Helix C6 of the C-terminal domain of gp96 forms disordered straps to give stability to the gp96 dimer. In our result, since GPM1 confers slight helical change of gp96 (Fig. S2C) and dimer/oligomer formation of gp96 (Fig. 2G–I), we speculate that GPM1 stabilizes disordered helix C6 straps of the C-terminal domain of gp96 to lead gp96 dimer formation.

SLE is characterized by abnormalities in dendritic cell (DCs), autoreactive T cells and B cells [1],[2]. DCs are important in regulating both immunity and tolerance and have been implicated in the pathogenesis of SLE [1]. DCs induce naïve T cell activation and stimulate B cell growth and differentiation. The lupus-associated DCs producing altered signals amplify autoreactive specificities in T cells, which, in turn, stimulate autoreactive B cells, leading to the increase of autoantibodies. Since cell surface gp96 induced significant DC activations [29],[32], we employed a chemical method to intervene over-maturation of DCs via gp96. The results of this work consistently show that aberrant surface exposure of gp96 can be chemically controlled. The selective suppression of cell surface gp96 reduced the incidence and severity of SLE-associated phenotypes, suggesting gp96 as a potential target to control autoimmune disease like SLE.

## Materials and Methods

### Drug Administration

Female gp96tm transgenic mice were bred and maintained at the animal center for pharmaceutical research, Seoul National University. We used age-matched, 12∼26 weeks old mice in each experimental group. To suppress cell surface gp96 we used GPM1 compound. Dexamethasone and GPM1 were dissolved in 5% DMSO in phosphate-buffered saline (PBS) and control vehicle was 5% DMSO in PBS. Compounds were administered i.p. every 24h for 2 months (30mg/kg/day). Three groups of each 9 mice were examined. All procedures were conducted in accordance with the *Guide for the Care and Use of Laboratory Animals*, published by the Korean National Institute of Health. This study was approved by Review Board of Institute of Laboratory Animal Resources of Seoul National University.

### Flow Cytometry

Spleen and lymph node cell suspensions were prepared by grinding tissue through sterile mesh and erythrocytes were lysed with RBC lysis buffer (eBioscience, CA). For surface staining, antibodies were fluoresceinisothiocyanate (FITC)-, phycoerythrin (PE)-, PerCP-, or APC-conjugated. Antibodies used were CD4 (RM4-5, H129.12), CD8 (53-6,7), B220 (RA3-6B2), CD11b (M1/70), CD11c (HL3), CD44 (IM7), CD62L (MEL-14), CD69 (H1.2F3), CD25 (PC61.5), IgM (11/41), IgD (11-26C.2a), calreticulin (BD Pharmingen, CA), MHC class II (M5/114.15.2), Foxp3 (FJK-16s) (eBioscience, CA), gp96(Santacruz, CA), and PDI (Assay Designs, MI). After staining, cells were washed and analyzed on a FACScan flow cytometer using CellQuest software (BD Bioscience, Mountain View, CA).

### Mixed Lymphocyte Reaction

Splenic DCs (8×10^3^) from vehicle- or GPM1-treated mice were purified using CD11c magnetic beads (Miltenyi Biotec, Auburn, CA), fixed with 1% paraformaldehyde, and co-cultured with 1.5×10^5^ purified allogeneic CD4^+^ T cells. Culture supernatants were collected for TNFα assay using ELISA kit from Pierce Biotechnology Inc. (Rockford, IL).

### ELISA Assay for Chemical Screening

96-well plates (Maxisorp., F96; Nunc) were coated with 500ng/well AIMP1in PBS (pH 7.4). After washing, the remaining sites were blocked with PBS containing 1% BSA for 1h. Binding of 10ng/well biotin-conjugated gp96 was performed in Tris buffer (25 mM Tris, 10 mM NaCl and 0.4% Triton X-100). Each of the 6,580 compounds was added to the well at 100nM and the plates were washed and incubated with HRP-conjugated streptavidin in PBS containing 0.1% BSA and 0.1% Tween 20 for 30 min. The plates were washed, and then substrate was added to each well. The absorbance was monitored at 450 nm.

### Histology

Kidney tissue sections of gp96tm transgenic mice were fixed in 10% formaldehyde in PBS and dehydrated using an alcohol gradient. After paraffin infiltration, the tissues were sectioned using a microtome, stained with hematoxylin and eosin (H&E) and analyzed by light microscopy. For immunofluorescent staining, kidneys were sectioned with a cryostat. These cryosections were blocked with normal goat serum, stained with FITC-conjugated goat anti-mouse Ig (BD pharmingen, CA), and then observed by fluorescent microscopy. Glomerulonephitis was quantitated according to Berden scores [42].

### Surface Plasmon Resonance (SPR)

Human gp96 was expressed as His tag fusion protein in *Escherichia coli* BL21 (DE3) and purified by nickel affinity chromatography. His-tagged human gp96 was immobilized onto CM5 sensor chips using standard amine coupling^2^. PBS was used as a running buffer. The carboxymethyl dextran surface within one side of the flow cell was activated with a 7-min injection of a 1∶1 ratio of 0.4M EDC and 0.1M NHS. The protein was coupled to the surface with a 7-min injection of gp96 diluted in 10mM sodium acetate, pH 3.7. Remaining activated groups were blocked with a 7-min injection of 1.0M ethanolamine, pH 8.5. GPM1 compound was dissolved directly in the PBS running buffer containing 1% DMSO and injected at a flow rate of 20µl/min at 25°C and the binding was determined by the change in resonance units (RU). The compound concentration varied from 10nM to 25µM and each concentration was tested at least three times. All of the bound complexes dissociated back to baseline within a reasonable time frame; therefore, no regeneration was required. Sensorgram was processed by subtracting the binding response recorded from the control surface, followed by subtracting an average of the buffer blank injections from the reaction spot^3^. To determine kinetic rate constants, all data sets were fit to a simple 1∶1 binding with drifting baseline model using BIAevaluation program.

### Purification of gp96

His-tagged gp96 protein was expressed in *E. coli* BL21 (DE3) and purified by nickel affinity chromatography and by gel filtration chromatography, and dialyzed against buffer (25mM Tris-HCl, pH7.5, 50 mM NaCl).

### Circular dichroism (CD)

The gp96-GPM1 complex was prepared at a molar ratio 1∶10 (5µM∶50µM) in 250µl of buffer (25mM Tris-HCl, pH7.5, 50mM NaCl). The final concentration of DMSO was 0.1%. The complex was incubated at 20°C for 1h before CD measurements were taken. Samples were loaded in a 0.2 cm CD cell, and the spectra were acquired at 20°C using Jasco J-810. Wavelength scans were performed between 195 and 260 nm. The signal was digitized at 1nm intervals and each final scan was an average of 3 repeats and is presented after baseline buffer correction.

### FPLC of gp96

FPLC gel filtration was used to separate gp96 monomer, dimer, and oligomer. gp96 or gp96-GPM1 complex containing a 1∶10 molar ratio of gp96 to GPM1 were injected into a Superdex 200 10/300 GL (GE healthcare, 10 mm i.d. ×30 cm length) size exclusion column. The elution (0.5 ml/min) was carried out with buffer (20mM KH_2_PO_4_, 500mM NaCl, pH7.8, 2mM β-mercaptoethanol, 300mM imidazole). Fractions of a volumn of 0.5 ml were collected during 60 min.

### Statistical analysis

The student's *t*-test was used for statistical analysis. *P* values of <0.05 were considered to represent statistically significant differences.

## Supporting Information

Figure S1 The binding of GPM1 to protein disulfide isomerase (PDI) (A) and GRP78/Bip (B) were examined by surface plasmon resonance (SPR). GPM1 at the indicated concentrations was injected to immobilized PDI and GRP78/Bip, and the binding was measured using Biacore 3000. The response data were processed using data from a reference surface and buffer injections. Equilibrium dissociation constant (KD) was determined for the interaction of GPM1 with PDI and GRP78/Bip.
(0.28 MB TIF)

Figure S2 The effect of point mutations on the ATPase activity of gp96. The effect of gp96 point mutations on its ATPase activity was examined using ATPase assay kit (Innova Biosciences, Cambridge, UK). One unit is the amount of enzyme that catalyzes the reaction of 1µmol substrate per minute. The enzymatic activity was calculated according to manufacturer's instruction.
(0.10 MB TIF)

Figure S3 The effect of GPM1 on gp96 ATPase activity and conformational change. (A) The dose-dependent effect of GPM1 on the ATPase activity of gp96. The gp96 activity in the absence of GPM1 was 0.02066±0.00334 unit/ml and GPM1 showed little effect on the ATPase activity within the range of the tested concentration (0 vs. 100µM, p = 0.071). (B) Circular dichroism spectrum of free and GPM1-bound gp96 protein. GPM1 (50µM, >95% purity) was mixed with gp96 (5µM). The CD spectra were normalized by buffer containing 0.1% DMSO.
(0.16 MB TIF)

Table S1 Summary of the screening of the chemicals derived from the primary hits that inhibit the interaction between gp96 and AIMP1 more than 95% of the control at 0.1µM.
(0.19 MB TIF)

Table S2 Comparison of glomerulonephritis in gp96tm transgenic mice treated with vehicle (n = 9), GPM1 (n = 9), or dexamethasone (n = 7). Glomerulonephitis was quantitated according to Berden scores ^26^.
(0.14 MB TIF)
