# Supplementary figures and images for: Identification of gp96 as a Novel Target for Treatment of Autoimmune Disease in Mice
Source: PLoS One. 2010 Mar 23;5(3):e9792. doi: 10.1371/journal.pone.0009792 (PMC2843739; doi:10.1371/journal.pone.0009792)

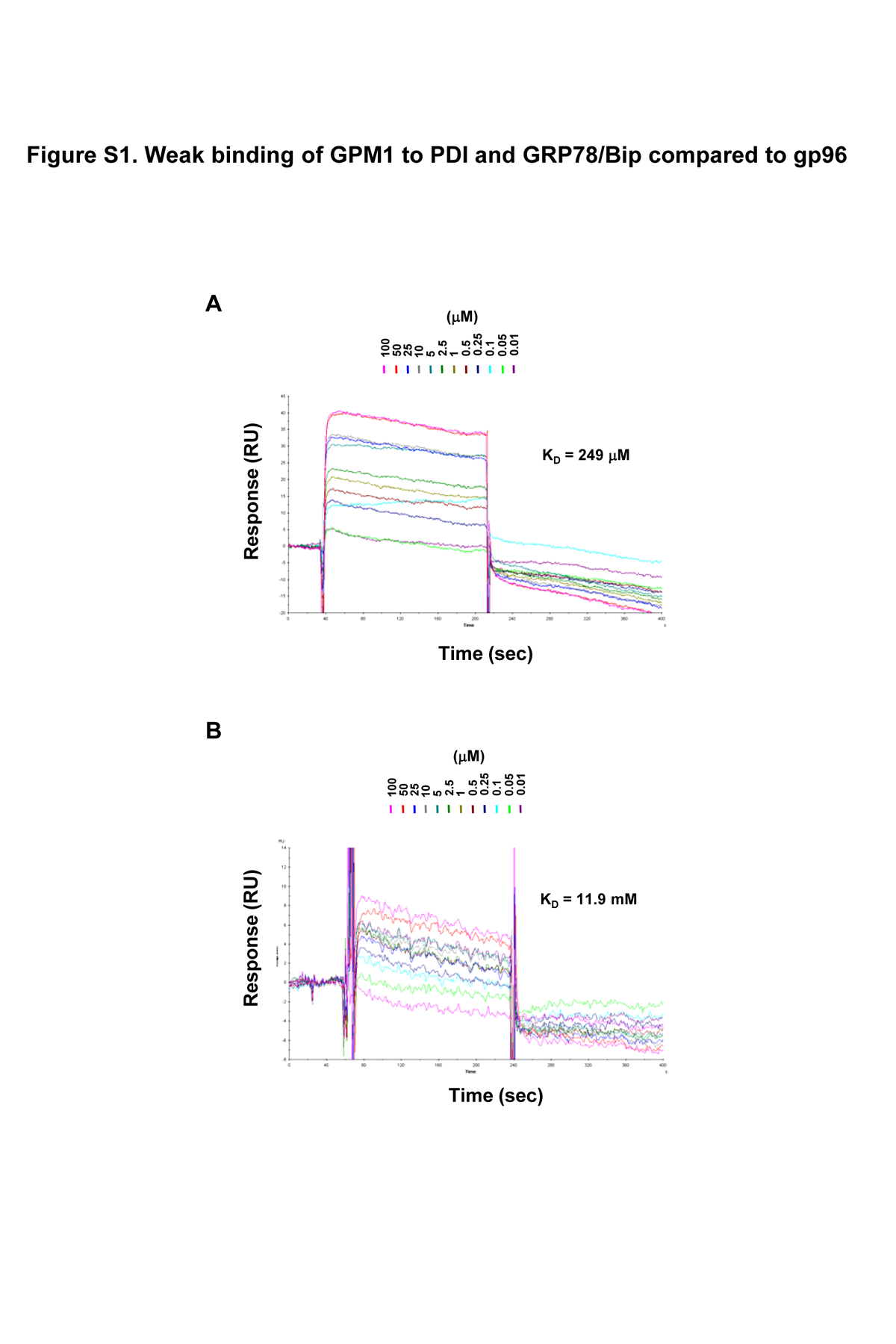

Supplement: Figure S1 — The binding of GPM1 to protein disulfide isomerase (PDI) (A) and GRP78/Bip (B) were examined by surface plasmon resonance (SPR). GPM1 at the indicated concentrations was injected to immobilized PDI and GRP78/Bip, and the binding was measured using Biacore 3000. The response data were processed using data from a reference surface and buffer injections. Equilibrium dissociation constant (KD) was determined for the interaction of GPM1 with PDI and GRP78/Bip. (0.28 MB TIF) [file pone.0009792.s001.tif]

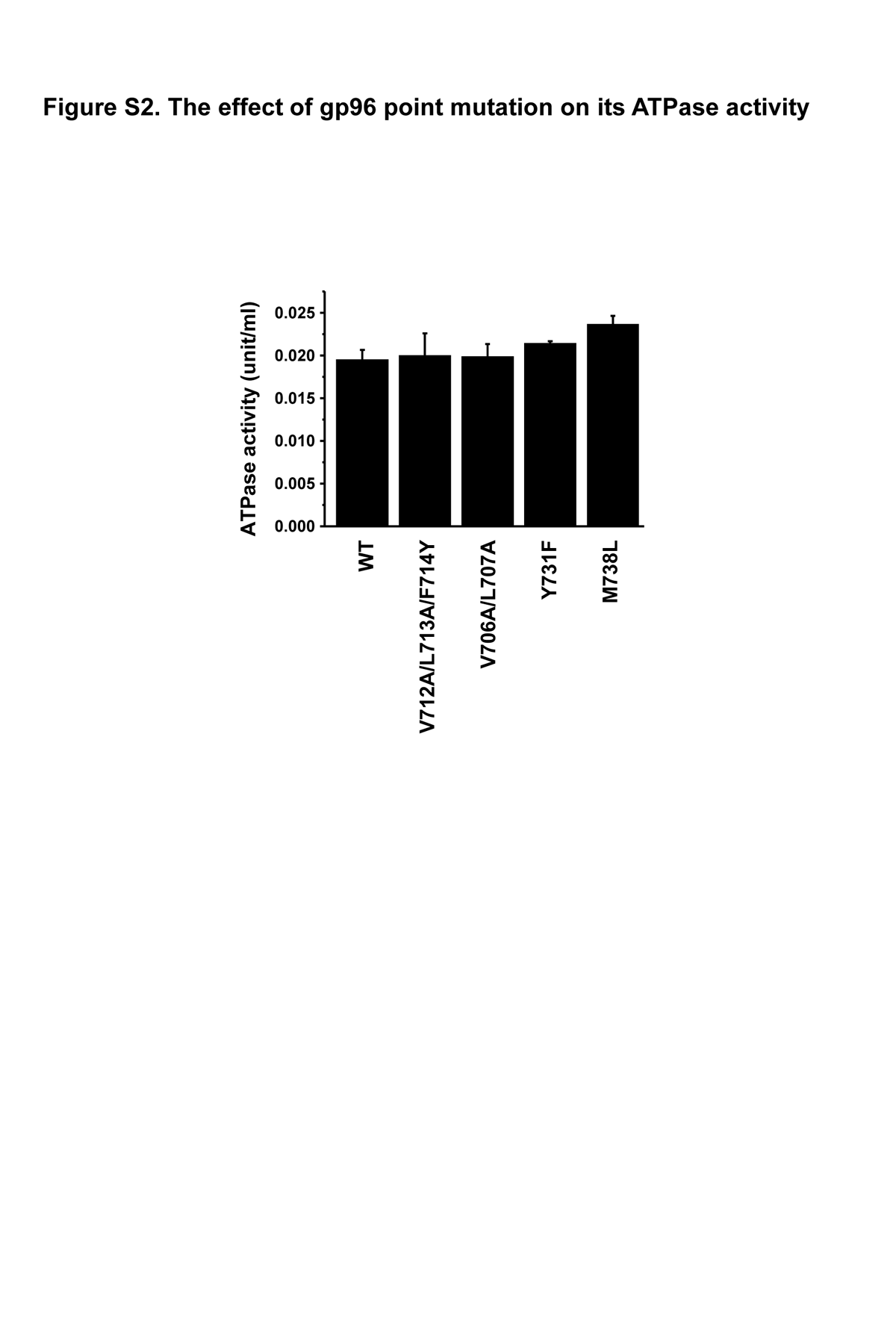

Supplement: Figure S2 — The effect of point mutations on the ATPase activity of gp96. The effect of gp96 point mutations on its ATPase activity was examined using ATPase assay kit (Innova Biosciences, Cambridge, UK). One unit is the amount of enzyme that catalyzes the reaction of 1µmol substrate per minute. The enzymatic activity was calculated according to manufacturer's instruction. (0.10 MB TIF) [file pone.0009792.s002.tif]

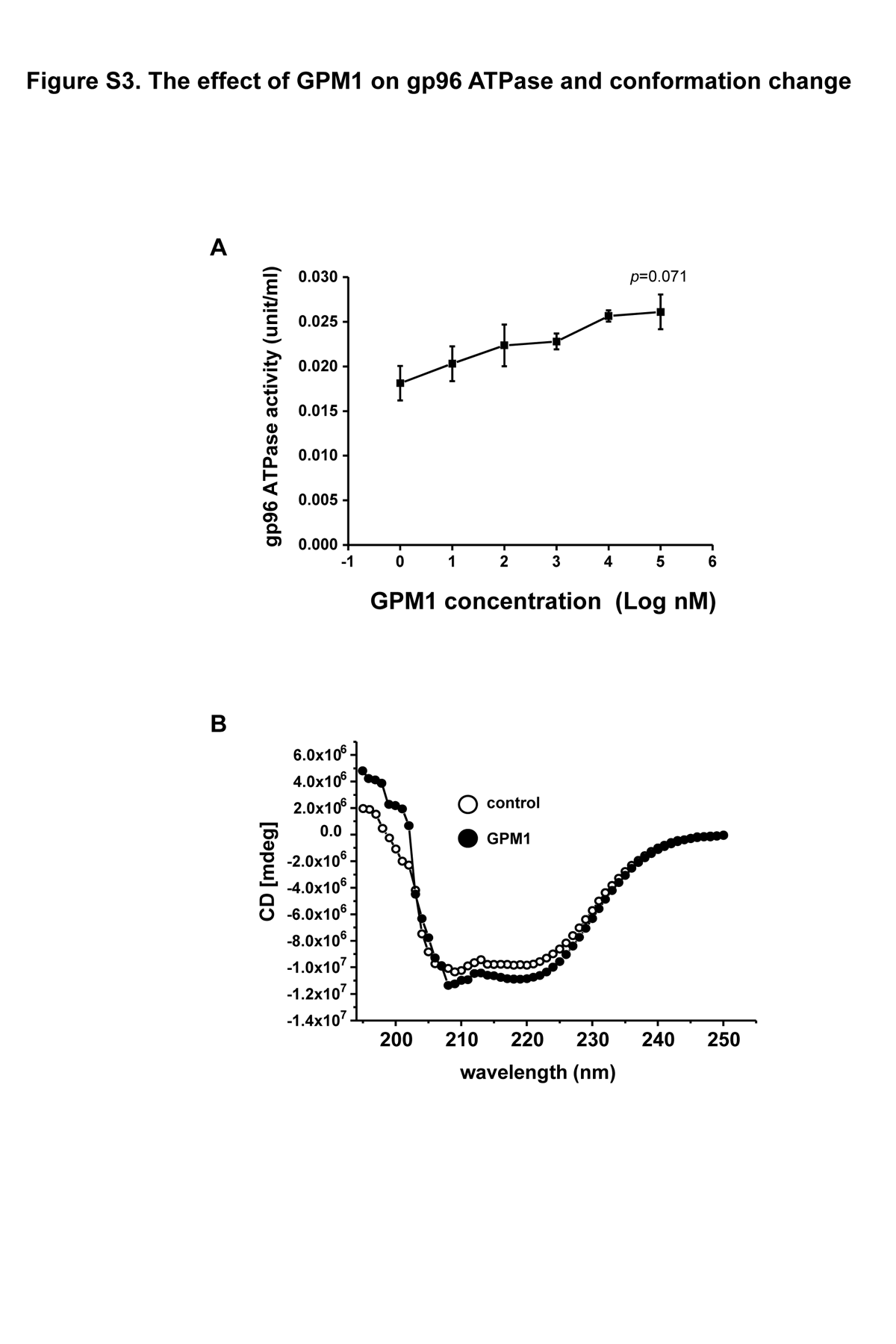

Supplement: Figure S3 — The effect of GPM1 on gp96 ATPase activity and conformational change. (A) The dose-dependent effect of GPM1 on the ATPase activity of gp96. The gp96 activity in the absence of GPM1 was 0.02066±0.00334 unit/ml and GPM1 showed little effect on the ATPase activity within the range of the tested concentration (0 vs. 100µM, p = 0.071). (B) Circular dichroism spectrum of free and GPM1-bound gp96 protein. GPM1 (50µM, >95% purity) was mixed with gp96 (5µM). The CD spectra were normalized by buffer containing 0.1% DMSO. (0.16 MB TIF) [file pone.0009792.s003.tif]

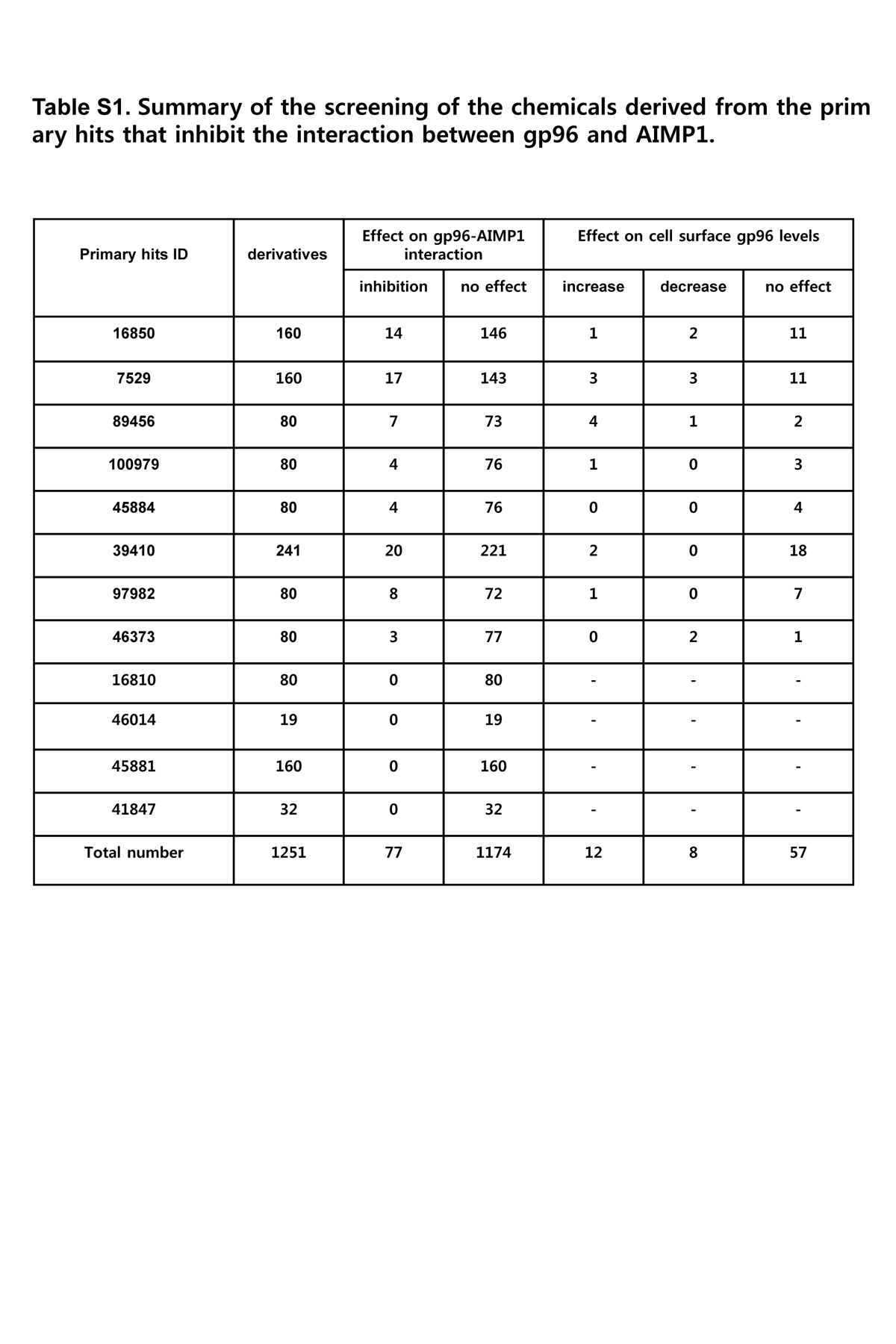

Supplement: Table S1 — Summary of the screening of the chemicals derived from the primary hits that inhibit the interaction between gp96 and AIMP1 more than 95% of the control at 0.1µM. (0.19 MB TIF) [file pone.0009792.s004.tif]

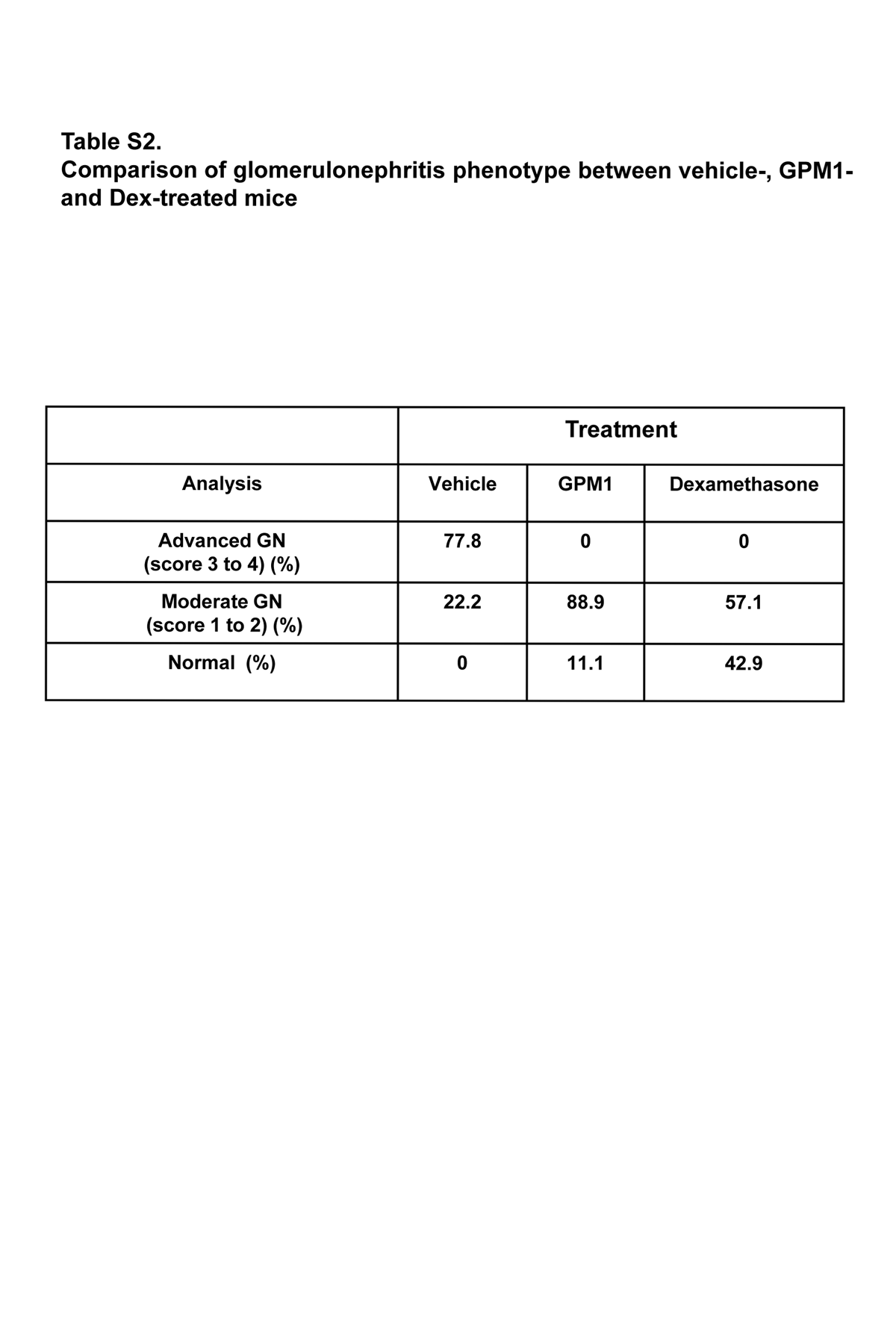

Supplement: Table S2 — Comparison of glomerulonephritis in gp96tm transgenic mice treated with vehicle (n = 9), GPM1 (n = 9), or dexamethasone (n = 7). Glomerulonephitis was quantitated according to Berden scores 26. (0.14 MB TIF) [file pone.0009792.s005.tif]
